# Molecular Features of HPV-Independent Cervical Cancers

**DOI:** 10.3390/pathogens14070668

**Published:** 2025-07-08

**Authors:** Luca Giannella, Camilla Grelloni, Leonardo Natalini, Gianmarco Sartini, Mila Bordini, Giovanni Delli Carpini, Jacopo Di Giuseppe, Erica Dugo, Francesco Piva, Andrea Ciavattini

**Affiliations:** 1 Gynecologic Section, Woman’s Health Sciences Department, Polytechnic University of Marche, 60123 Ancona, Italy; luca.giannella@ospedaliriuniti.marche.it (L.G.); c.grelloni@pm.univpm.it (C.G.); l.natalini@pm.univpm.it (L.N.); g.sartini@pm.univpm.it (G.S.); m.bordini@pm.univpm.it (M.B.); giovanni.dellicarpini@ospedaliriuniti.marche.it (G.D.C.); jacopo.digiuseppe@ospedaliriuniti.marche.it (J.D.G.); 2 Department of Specialistic Clinical and Odontostomatological Sciences, Polytechnic University of Marche, 60131 Ancona, Italy; e.dugo@pm.univpm.it (E.D.); f.piva@univpm.it (F.P.)

**Keywords:** cervical cancer, HPV-independent, molecular features

## Abstract

HPV-independent cervical cancers represent a small proportion of these types of cancers, predominantly glandular lesions. It should be noted that some cases may depend on diagnostic problems that lead to false negative cases. However, the most recent classifications distinguish cervical tumors into HPV-associated and HPV-independent cancers. HPV-negative cervical carcinomas (5–11% of all cases) mainly include rare adenocarcinomas (gastric, mesonephric, clear, serous, and endometrioid) and present distinct clinical and molecular features. These tumors usually affect older women and are diagnosed at more advanced stages than HPV-positive tumors, with an overall worse prognosis. This concerning and notably worse prognosis highlights the need for further research and understanding. Unlike HPV-positive carcinomas (which depend on the viral oncogenes E6/E7), HPV-independent tumors accumulate genomic mutations that activate oncogenes and inactivate suppressor genes. Therefore, a comprehensive overview of these aspects can be the key to a better understanding and developing personalized treatments. In the present review, the main mutated genes, the signaling pathways involved, the differences from HPV-positive tumors, the distinctive immunohistochemical markers, and the diagnostic and therapeutic implications are explored in depth.

## 1. Introduction

HPV-negative cervical carcinomas are rare since they account for approximately 5% of all cervical carcinomas [1,2,3,4,5]. However, their relative prevalence is higher among cervical adenocarcinomas (which account for approximately 20–25% of all cervical cancers): in this group, approximately 5–10% are not HPV-related [6,7,8]. The most representative example is gastric-type adenocarcinoma (GAS), which accounts for ~10% of cervical adenocarcinomas globally [9]. These neoplasms preferentially affect older women: the average age of diagnosis is around 50–55 years (generally much higher than in patients with HPV-positive tumors) [10].

In many cases, the diagnosis occurs in advanced stages because of the lack of early signs and symptoms in HPV-negative tumors. Interestingly, with the widespread adoption of the anti-HPV vaccine, the relative share of HPV-independent adenocarcinomas is likely to increase [6]. This category encompasses both rare squamous carcinomas and several adenocarcinoma subtypes, all of which lack the viral oncoproteins E6/E7. Indeed, these neoplasms typically exhibit a lack of p16 tumor suppressor gene overexpression (often negative or only focal), and their growth depends on distinct oncogenic pathways [6]. The WHO 2020 classification distinguishes HPV-associated cervical histotypes from HPV-independent ones (Table 1) [11,12]. From a molecular point of view, the absence of E6/E7 means that HPV does not degrade the cellular genes TP53 and RB; therefore, mutations of TP53 and other tumor oncosuppressor genes (e.g., STK11, PTEN) and oncogenes (such as KRAS, PIK3CA, and ARID1A) are frequently detected in HPV-negative tumors [13]. More specifically, mutations in TP53 in the core domain (Y103G, Y107G, T155D, L265A) can inhibit the interaction between E6 and p53, preventing E6 from targeting p53 for degradation.

From a diagnostic perspective, there are considerable difficulties. HPV-negative tumors escape standard screening tests: both Pap tests and HPV tests are frequently regular or false negative [9]. Furthermore, HPV-independent carcinomas tend to have a worse prognosis than HPV-positive ones [14,15,16,17]. This is partly due to the late diagnosis (resulting from a lack of screening signals) and the aggressive behavior of specific subtypes (e.g., GAS and CCC). GAS and clear cell adenocarcinomas (CCC) are often discovered at advanced stages and show worse survival rates [13]. Currently, there are no specific therapeutic protocols based on HPV status. The treatment (surgery, chemotherapy, or radiotherapy) is primarily driven by the stage of the disease and its pathologic features, such as HPV positivity or negativity. However, tumor-specific molecular biology may enable the development of targeted strategies. [10,18].

The topic’s importance is evident from the above. In this review, we focus on the primary molecular alterations of these tumors, the molecular signaling pathways, the differences between these tumors and HPV-related tumors, the immunohistochemical characteristics, and potential targeted therapies.

## 2. Critical Issues in Identifying HPV-Independent Cervical Cancers: False Negatives

False negative HPV tests constitute a significant issue in diagnosing cervical cancer, especially in cases suspected to be HPV-negative. Here is a closer look at the reasons and implications of these false negatives [3,19,20,21,22,23,24], which can be substantially divided into three conditions:

**Test-Specific Technical Issues**

- Standard tests (such as PCR or in situ hybridization) may not detect HPV types not included in the test panel (rare or classic non-oncogenic variants).

- The test may not detect a low viral load due to its detection limit.

**Sample Quality**

- In biopsy samples preserved in formalin, HPV DNA degradation may reduce the sensitivity of the molecular test.

- Insufficient sampling, suboptimal storage, or errors in the transport or analysis phase may compromise the result.

**Biological Implications**

- Integration of viral DNA into the human genome may be partial or involve the loss of regions targeted by the test (e.g., L1 gene), making the virus undetectable.

Some cervical tumors, especially some subtypes of adenocarcinoma such as GAS, mesonephric adenocarcinoma (MNAC), and CCC, are biologically HPV-independent and, therefore, authentically HPV-independent tumors, despite their cervical origin.

Therefore, when we are facing HPV-independent cervical cancer, all these eventualities should be taken into consideration.

## 3. Main Histological Histotypes

HPV-negative cervical cancer mainly includes specific subtypes of adenocarcinoma. The main histotypes (all excluded by viral infection) are:

- **Gastric (Gastric-Type) Adenocarcinoma (GAS):**

*Epidemiology:* It is the most common HPV-independent adenocarcinoma. It accounts for approximately 10% of cervical adenocarcinomas [25]. It affects women with an average age of 50–52 years [26].

*Clinical features:* Often, the tumor extends deep into the cervix without forming an obvious nodule, sometimes causing watery discharge but escaping conventional screening controls [26].

*Pathology:* The tumor cells appear tall and columnar with vacuolated or clear mucinous cytoplasm and show markers of gastric differentiation (e.g., MUC6 positive) [27]. GAS is particularly insidious: in well-differentiated forms (malignant adenoma or minimal-deviation adenocarcinoma), the nuclei are very little atypical, and vaginal cytology may be normal in 30–50% of cases [28,29]. The immunophenotype is characterized by CK7 + /MUC6 + expression, while p16 is typically negative or weakly expressed [6].

- **Mesonephric Adenocarcinoma (MNAC):**

*Epidemiology:* sporadic tumor (<1% of cervical adenocarcinomas) [30,31]. The average age of onset is ~52 years [32].

*Clinical features:* It often develops in the lateral-to-posterior area of the cervix. Typically, it does not form a well-defined mass and exhibits various growth patterns, including invasive, bulky, or exophytic [31]. It is an aggressive tumor: about 70% of cases are already in stage IB at diagnosis, with recurrences in about 30% of patients and early distant metastases [32,33,34].

*Pathology:* It originates from the embryonic remnants of the mesonephric (Wolffian) duct in the upper third of the cervix. Under the microscope, it reveals small, narrow tubules with eosinophilic secretions and cuboidal cells; these cells are PAX8+ and CD10+ and are typically negative for ER, PR, and CEA [35].

*Significant mutations:* In this type, an activating mutation of KRAS is almost always found (less frequently in NRAS); PIK3CA or PTEN mutations are not typically observed [35].

- **Clear Cell Adenocarcinoma (CCC):**

*Epidemiology:* It represents about 2–7% of cervical adenocarcinomas and is almost always HPV-independent (70–75% of cases are HPV-negative) [20,36].

*Clinical features:* It is historically associated with intrauterine exposure to DES; in the group of DES-exposed women, the peak incidence occurs very early (≈approximately 19 years). Clinically, cervical CCC is aggressive: the sensitivity of the Pap test is very low (only ≈18% of patients are positive), and high rates of local recurrence and distant metastases are observed (up to 50% in FIGO II tumors) [37,38,39]. In non-DES-related forms, the age of onset is more variable, while in DES-exposed patients, it can arise at a young age [40,41,42].

*Pathology:* The microscopic morphology resembles ovarian and uterine CCC: epithelial cells with abundant clear cytoplasm rich in glycogen and sometimes barley, sometimes with hyaline bodies. The typical immunophenotype is positive for HNF1β, Napsin-A, and PAX8, while ER and PR are negative [35,43]. The expression of p16 in this tumor can vary from negative to focal.

Table 2 summarizes the primary clinical, pathological, and molecular characteristics of HPV-independent adenocarcinomas.

## 4. Significant Mutations in HPV-Negative Cervical Cancers

Recent studies have unveiled the intricate genetic landscape of HPV-independent cervical carcinomas, revealing recurrent mutations in numerous oncogenes and suppressor genes. This complexity, particularly in the following genes, has sparked significant interest:

**- TP53** (tumor suppressor, p53 protein): mutated in approximately 40–50% of HPV-negative cervical GAS [10,18]. Mutation of TP53, normally inactivated by the virus in HPV-positive tumors, is a key event in HPV-negative tumors [29,44].

**- PIK3CA** (oncogene of the PI3K/AKT pathway): mutated in various subtypes of cervical adenocarcinoma; plays an important role in activating the PI3K/AKT/mTOR pathway [14,15,16,17]. In HPV-negative tumors (e.g., in some clear cell or serous tumors), PIK3CA mutations are present but are less frequent than in HPV-positive tumors [45].

**- KRAS** (RAS/MAPK oncogene): mutated in numerous mucinous cervical adenocarcinomas, including approximately 18–36% of GAS [10,18,26]. KRAS mutations (along with NRAS) are prevalent in HPV-independent MNAC [26].

**- ARID1A** (SWI/SNF complex for chromatin remodeling): Mutated in ~20–30% of GAS and many MNAC [18,45]. Alterations in ARID1A (and other SWI/SNF subunits such as ARID1B and SMARCA4) contribute to the HPV-independent transformation of cervical epithelial cells. The SWI/SNF complex regulates gene expression by altering chromatin structure, and mutations in its subunits can lead to dysregulation of gene expression, contributing to cancer development [26].

**- PTEN** (PI3K/AKT pathway tumor suppressor): lost or mutated in approximately 20% of GAS; loss of PTEN has also been reported in cervical CCC, with subsequent activation of AKT [45,46]. The loss or mutation of PTEN in these cancers leads to the activation of the PI3K/AKT pathway, which promotes cell survival and growth, making PTEN a potential therapeutic target.

**- STK11** (LKB1) (tumor suppressor): mutated in 33–55% of GAS (including minimally differentiated mucinous carcinomas, MDA) [10,18,45]. STK11 mutations, or Peutz-Jeghers syndrome, are characteristic of HPV-independent gastric and cervical subtypes [10,18,45].

**- CDKN2A** (p16 gene): mutated in 18–27% of GAS [10,18]. CDKN2A encodes the p16 protein (IHC marker), implicated in cell cycle control. In HPV-negatives, CDKN2A alterations contribute to mitotic cycle dysregulation.

**- ERBB2** (HER2): amplified in approximately 13% of GAS; HER2 overexpression has been observed in ~30% of HPV-negative cervical serous carcinomas [10,18,45,47]. Alterations in ERBB2/ERBB3 are potential targets for anti-HER2 therapies.

Other less common but reported genes include mutations in FGFR2 (in some mesonephric tumors), BRCA2, BRAF, NTRK3, and DNA repair genes (e.g., POLE). For example, isolated cases of CCC have shown mutations in the POLE gene, which is associated with a high mutational burden. These mutations involve critical pathways of carcinogenesis (cell cycle, cell growth, DNA repair, and chromatin remodeling), as outlined in the following section [26].

## 5. Molecular Signaling Pathways

In HPV-negative cervical tumors, neoplastic transformation is mainly based on genomic mutations that activate oncogenes, inactivate suppressor genes, or involve chromatin remodeling and DNA repair:

**Oncogenes Activation:**

- **PI3K/AKT/mTOR Pathway**—often activated by PIK3CA mutations or loss of PTEN, with hyperactivation of AKT/mTOR; this pathway promotes cell proliferation and survival [48,49]. For example, in CCC, expression of p-AKT and p-mTOR is observed in approximately 50% of cases [46].

- **RAS/MAPK Pathway**—KRAS/NRAS mutations activate the RAS→RAF→MEK→ERK cascade, which is frequently observed in mesonephric and mucinous subtypes [50,51,52,53]. RAS/MAPK pathway inhibitors are being studied in gynecological oncology [54].

- **Growth Factor Signaling Pathways**—Amplifications of tyrosine kinase receptors (e.g., ERBB2, FGFR2, EGFR) promote proliferation via PI3K/AKT and RAS/MAPK pathways. E.g., HER2 amplified in GAS or serous carcinomas [10,18,45].

**Inactivation of Oncosuppressor Genes:**

- **Cell Cycle Control**—Inactivation of TP53 (mutation) prevents cell cycle arrest after DNA damage; CDKN2A/p16 mutations disable the G1 checkpoint. In HPV-positive tumors, these checkpoints are compromised by the viral oncoproteins E6 and E7, whereas in HPV-independent tumors, the effect is due to genomic mutations [29,44,55].

**Chromatin Remodeling Deregulation:**

- **Mutations in SWI/SNF Chromatin Remodeling Complexes (ARID1A, ARID1B, SMARCA4, and BCOR)**—lead to epigenetic deregulation of genes; present especially in GAS and mesonephric subtypes [26].

**DNA Repair Pathways:**

- **MMR**—Some HPV-negatives (particularly CCC) show microsatellite instability. Deficiencies in mismatch repair (MMR) genes or mutations in POLE are detected, resulting in genomic instability [13,43]. These alterations can stimulate immune responses and influence sensitivity to immunotherapies.

- **Homologous Recombination Repair (HRR)**—The main HRR genes include BRCA1 and BRCA2, ATM, ATR, PALB2, and SLX4. In HPV-independent cervical cancers, these genes are rarely mutated overall; however, in some histotypes (adenocarcinoma gastric type), BRCA2 has been found mutated in approximately 21% of cases, and SLX4 in 36% of cases [19]. This data may be of interest since HRR-deficient cervical cancers can be targeted by PARP inhibitors [56].

- **Base Excision Repair (BER) and Nucleotide Excision Repair (NER)**—Genes involved in these pathways, such as ERCC2, XPA, and XRCC1, may be altered, although very rarely and without clear clinical implications in HPV-independent cervical cancers [57,58].

**Other Pathways:**

Recent studies also highlight the involvement of pathways such as WNT [mutations in CTNNB1 in endometrioid adenocarcinomas (EAC)] and TGF-β (regulated by lncRNAs such as NEF in HPV-negatives) [27,59].

**Figure 1:**
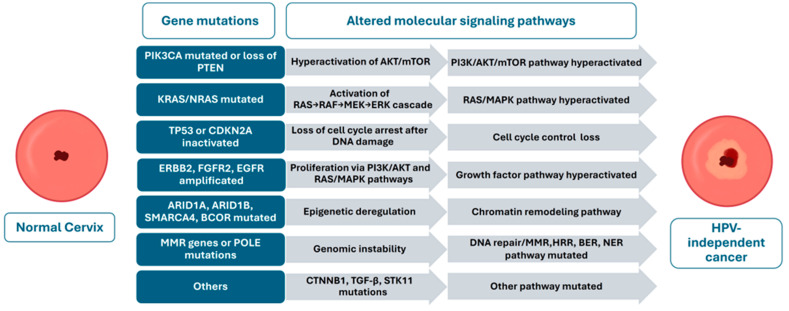
Gene mutations and molecular signaling pathways involved in the malignant trans-formation of HPV-independent cervical cancers.

Figure 1 schematically summarizes the process of genetic mutations and altered molecular signaling pathways that lead to HPV-independent cervical cancers.

## 6. Molecular Differences Compared to HPV-Positive Tumors

HPV-positive cervical tumors, whether squamous or adenocarcinomas, are notable for their dependence on the integration of the oncogenic viral genomes E6/E7. This unique characteristic sets them apart from HPV-negative tumors, which develop independently of HPV. The main differences are:

- **Oncogenic mechanism**: In HPV-positive tumors, viral proteins degrade p53 and pRb, leading to hyperactivity of p16. Conversely, HPV-negative tumors do not contain viral DNA and have direct mutations in cell cycle genes. Consequently, the p53 protein is mutated and stable in HPV-negative tumors, while in HPV-positive tumors, p53 is destroyed by E6 [18,55]. Similarly, p16 levels are high in HPV-positive tumors (diffuse “block” pattern) but are absent or only focal in HPV-negative tumors [60,61,62,63];

- **Histology and histogenesis:** The HPV-positive tumors, typically squamous carcinomas or usual endocervical adenocarcinomas, are a testament to the intricate nature of these tumors. They are rich in “floating” apoptotic mitoses, a feature not commonly found in HPV-negative tumors. The latter include rare subtypes such as GAS, MNCA, CCC, serous, and EAC, which do not present the typical HPV-related morphological features [11,12,60,61,62,63];

- **Mutational profile:** As mentioned, HPV-negative tumors accumulate multiple mutations in TP53, KRAS, ARID1A, etc., while HPV-positive tumors have a mutational load often related to APOBEC but with fewer typical driver mutations [50,51,52,53,64]. These differences in mutational profiles have significant implications for diagnosis and treatment. For example, TP53 and PIK3CA are significantly more mutated in HPV-independent tumors than in HPV+ tumors, reflecting different pathogenetic mechanisms. Understanding these differences can guide the selection of targeted therapies and the development of personalized treatment plans;

- **Epidemiology and prognosis:** HPV-negative carcinomas affect older women (mean ~62 years vs. ~49 years for HPV + ) and are often diagnosed at advanced stages (FIGO ≥II) with greater local invasiveness and lymph node metastasis [65,66]. The 5-year outcome is generally worse. This phenomenon is highlighted by the fact that HPV-independent GAS has a much lower survival compared to usual HPV-related adenocarcinomas [9];

- **Classification:** The latest WHO classification 2020 (IECC) explicitly separates endocervical tumors into HPV-associated and HPV-independent subtypes [11,12]. This reflects the fact that HPV-negative subtypes follow autonomous biological pathways and require different diagnostic and therapeutic criteria.

These molecular differences explain the importance of recognizing HPV-negative tumors as distinct entities and developing ad hoc diagnostic/therapeutic approaches.

From the above, the fundamental differences between HPV-related and HPV-independent cervical cancers can be summarized as follows:

- HPV-positive lesions are initially driven by the integration of the virus DNA into the host DNA with the inhibition of Rb/p53 by viral oncoproteins. Usually, they present distinctive characteristic biomarkers, such as p16 positivity. Their progression to invasive cancer involves the presence of precursor lesions (CIN1-CIN3). Finally, from a clinical perspective, they have a better prognosis due to a greater response to treatments.

- HPV-independent lesions instead involve somatic mutations of the host genes from the initial stages, not driven by HPV infection. They have different altered molecular pathways compared to their HPV+ counterpart and lack clear precursor lesions. Furthermore, they can escape primary and secondary prevention. Usually, the prognosis is worse because they have more aggressive behavior, and treatments are less effective.

**Figure 2:**
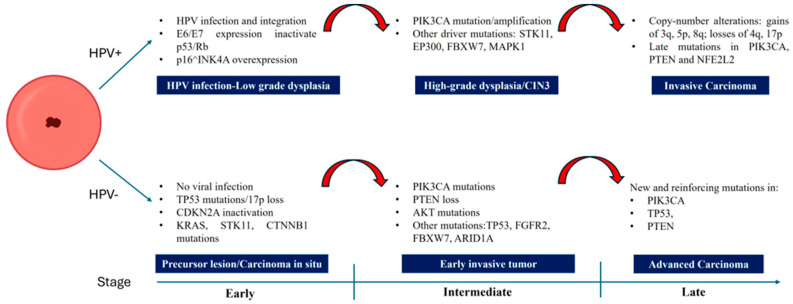
Main progression pathways through stages in HPV-related and HPV-independent cervical cancers.

Figure 2 shows the different stages of neoplastic transformation according to the main progression pathways in HPV- and non-HPV-related cervical cancers.

## 7. Distinctive Immunohistochemical Markers

In histopathological analysis, the use of immunohistochemical panels aids in distinguishing HPV-negative cervical carcinomas.

The key markers are as follows:

- **p16:** In HPV-positive tumors, the p16 protein shows diffuse “block” nuclear/cytoplasmic expression (E7-induced phenomenon). In contrast, in HPV-negative tumors, p16 expression is absent or only focal [60,61,62,63]. Therefore, a negative p16 IHC suggests an HPV-independent neoplasia;

- **p53:** In normal conditions, p53 has a scant pattern, but it accumulates in the nuclei in case of mutation. HPV-negative cervical carcinomas with TP53 mutations show marked nuclear expression (or marked absence) of p53 on IHC [29,44]. In HPV-positive tumors, p53 appears wild-type (absence of staining);

- **Gastric mucins (MUC6, HIK1083):** GAS express gastric mucin markers. In IHC, MUC6 and the HIK1083 antibody are positive in approximately 60–80% of these cases [60,67]. Positivity for these markers (e.g., CAIX) supports the diagnosis of the HPV-independent gastric subtype;

- **HNF1β, Napsin A:** typical of CCC, which diffusely express HNF1β and Napsin A, markers that distinguish them from other adenocarcinomas [35,43];

- **PAX8:** a protein typical of tubo-ovarian and endocervical neoplasms; rarely expressed in metastases of different origins. It is positively stained in many HPV-independent cervical carcinomas, confirming the cervical origin [35,43];

- **Hormone receptors and vimentin:** ER and PR are generally absent in HPV-negative GAS or MNAC, while vimentin expression is typical of endometrial adenocarcinomas. Cervical carcinoma without ER/PR and vimentin support cervical origin; conversely, diffuse positivity of ER/PR/vimentin suggests endometrial origin with spread to the cervix [9,36];

- **CEA, CD10:** The CEA marker tends to be low/negative in HPV-negative endocervical carcinomas, while CD10 is generally negative (often positive in endometrial adenocarcinomas). The combination of negativity for CD10 and CEA and the absence of HPV points to primary cervical carcinoma [9,36].

Below is a summary table with distinctive IHC markers in HPV-negative cervical tumors (Table 3):

## 8. Diagnostic Implications and Targeted Therapies

Knowledge of the molecular profile is crucial for differential diagnosis and the development of targeted therapies. In practice, for HPV-negative cervical tumors, metastasis or uterine involvement must be excluded: the combined use of histology and IHC (p16, p53, ER/PR, vimentin, CEA, CD10) helps to confirm the cervical origin [3,30,36]. For example, an endocervical GAS is distinguished from an endometrial adenocarcinoma by ER/PR/vimentin negativity, expression of gastric mucins (MUC6/HIK1083), and absence of CD10 [9,60,67].

On the therapeutic front, HPV-negative tumors currently have no specific options differentiated from the general cervical treatment guidelines. However, the recognition of mutations allows us to hypothesize precision therapies:

- **HER2-targeting:** in HPV-negative cervical serous carcinomas with HER2 overexpression (~30% of cases), the addition of trastuzumab to chemotherapy has been shown to extend survival by several months [13];

- **FGFR inhibitors:** In rare cases of MNAC with an FGFR2 mutation, treatment with tyrosine kinase (FGFR) inhibitors has obtained favorable clinical responses [33];

- **PI3K/AKT/mTOR inhibitors:** the presence of activating mutations of PIK3CA or loss of PTEN suggests the potential efficacy of drugs targeted against the PI3K/AKT/mTOR pathway (although their clinical use in the cervix is still experimental) [48,49];

- **Immunotherapy:** Tumors with a high mutational rate (e.g., POLE mutations, microsatellite instability) often express high levels of PD-L1 and present numerous neoantigens. In CCC, cases with high PD-L1 expression associated with POLE mutations have been described, making them potentially sensitive to anti-PD-1/PD-L1 checkpoint inhibitors [43,68,69,70]. Furthermore, CTNNB1 and other mutations may influence the immune microenvironment, although data on immunotherapy in HPV-negative tumors are still scarce;

- **Other targets:** BRAF or NTRK mutations (if present) open the way to specific therapies, such as BRAF inhibitors or TRK inhibitors. Similarly, mutated ARID1A is the subject of preclinical research (e.g., studies on EZH2 inhibitors or DNA repair checkpoint inhibitors) [9].

To summarize, the identification of mutated genes and key signaling pathways can promote the inclusion of patients in clinical trials with innovative therapies or combinations of targeted drugs. Finally, it should be noted that the adoption of molecular profiling-based strategies is still under investigation and requires integration with standard surgical, chemotherapy, and radiotherapy protocols.

**Figure 3:**
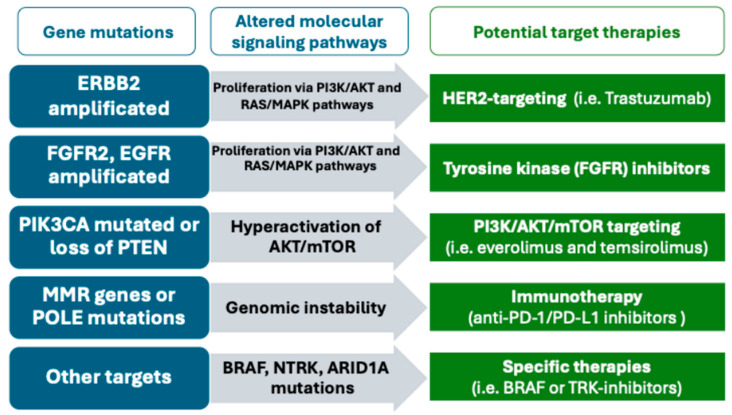
Gene mutations, molecular signaling pathways involved, and their potential target therapies.

Figure 3 shows potential target therapies for each genetic mutation.

## 9. Conclusions

HPV-independent cervical cancers, while uncommon, pose a significant diagnostic and therapeutic challenge. These cancers exhibit distinct gene mutations and altered molecular signaling pathways, distinguishing them from cancers associated with HPV. Unfortunately, their survival and disease recurrence prognosis are worse than the latter. However, we can gain a better understanding of their development and progression by focusing on their molecular features. Whenever a pathologist diagnoses an HPV-independent tumor, it is advisable to use panels to search for specific mutations that may direct the patient versus targeted therapies. Scientific research must explicitly characterize the effect of different mutations that can fall on the same gene. We often discuss the mutated gene, but we lack information regarding the specific mutation of the gene. Another emerging aspect, with the advent of single-cell RNA sequencing techniques, is the ability to identify which pathways are active in a single cell and the presence of cellular populations that can guide the course of the disease and predict resistance to therapies. This understanding can inform the development of targeted and personalized therapies, thereby improving clinical practice in the diagnosis and treatment of these tumors.

## Figures and Tables

**Table 1:** Classifications of adenocarcinoma of the uterine cervix.

IECC 2018/WHO 2020	
HPV-Associated (HPVA)	Non-HPV-Associated (NHPVA)
Usual type	Gastric Type
Villoglandular	Clear Cells
Mucinous, NOS	Mesonephric
Mucinous, intestinal	Endometrioid
Invasive stratified mucin-producing	
Micropapillary	
Serous-like	

**Table 2:** Main clinical features of HPV-independent cervical adenocarcinomas.

Clinical Features	HPV-Independent Cervical Adenocarcinomas		
	Gastric Type	Mesonephric Type	Clear Cell Type
**HPV Testing**	Negative	Negative	70% negative
**Frequency**	10% of adenocarcinomas	<1% of adenocarcinomas	2–7% of adenocarcinomas
**Age of Incidence (Average)**	50–52 years	52 years	19 years (DES-exposed), variable (non-DES-exposed)
**Clinical Features**	- It extends deep into the cervix without forming an obvious nodule - Watery discharge	- Latero-posterior location of the uterine cervix - Invasive, bulky, or exophytic growth	- Very low sensitivity of the Pap test (only 18%) - High rates of local recurrence and distant metastases
**Pathology Features**	- Tall and columnar cells, with vacuolated or clear mucinous cytoplasm, with gastric differentiation - MUC6 positive	- Small, narrow tubules with eosinophilic secretions and cuboidal cells - PAX8 and CD10 positive	- Epithelial cells with abundant clear cytoplasm rich in glycogen and sometimes barley, sometimes with hyaline bodies - HNF1β, Napsin-A, and PAX8 positive
**Main Genetic Mutations**	TP53, STK11, KRAS, PTEN, ARID1A, BRCA2, CDKN2A, ERRB2ampl	KRAS, NRAS, PIK3CA, PTEN	PI3K/AKT/mTOR, MMR, POLE

**Table 3:** Main distinctive IHC markers to distinguish HPV-negative from HPV-positive tumors.

**p16^INK4a**	p16 protein; expression blocked in HPV+ tumors; absent or focal in HPV-negative tumors.
**p53**	p53 protein; wild-type pattern in HPV+ tumors; in mutated HPV-negative tumors appears with nuclear overexpression (or absent)
**MUC6/HIK1083**	Gastric mucins; markers of gastric differentiation, expressed in ~60–80% of HPV-negative GASs.
**Ki-67**	Variable; lower in well-differentiated HPV negative. High and diffuse in HPV positive lesions.
**ER, PR, Vimentin**	Hormone receptors and vimentin; generally negative in HPV-negative gastric/mesonephric carcinomas (on the contrary positive in endometrial neoplasms).
**CD10**	Marker of endometrial origin; negative in HPV-negative cervical carcinomas (useful in the differential diagnosis with uterine metastases).
**CEA**	Often positive in gastric-type ADC. Variable in HPV positive tumors.

## Data Availability

Not applicable.

## Abbreviations

The following abbreviations are used in this manuscript:

AKT	Protein kinase B
APOBEC	Apolipoprotein B mRNA editing enzyme, catalytic polypeptide
ARID1A	AT-rich interactive domain-containing protein 1A
ARID1B	AT-rich interactive domain-containing protein 1B
BCOR	BCL6 corepressor
BRAF	v-raf murine sarcoma viral oncogene homolog B1
BRCA2	Breast cancer gene 2
CAIX	Carbonic anhydrase IX
CCC	Clear cell adenocarcinoma
CDKN2A	Cyclin dependent kinase inhibitor 2A
CEA	Carcinoembryonic antigen
CK7	Cytokeratin 7
CTNNB1	Catenin Beta 1
DES	Diethylstilbestrol
DNA	Deoxyribonucleic acid
DOAJ	Directory of open access journals
EAC	Endometroid adenocarcinoma
EGFR	Epidermal growth factor receptor
ER	Estrogen receptor
ERBB2	Erb-B2 receptor tyrosine kinase 2
ERBB3	ERb-B2 receptor tyrosine kinase 3
ERK	Extracellular signal-related kinase
EZH2	Enhancer of Zeste Homolog 2
FGFR	Fibroblast growth factor receptor
FGFR2	Fibroblast growth factor receptor 2
FIGO	International Federation of Gynecology and Obstetrics
GAS	Gastric (gastric-type) adenocarcinoma
HER2	Human epidermal growth factor receptor 2
HNF1β	Hepatocyte nuclear factor 1β
HPV	Human papillomavirus
HPV+	Human papillomavirus positive
HPVA	HPV-Associated
IECC	International endocervical criteria and classification
IHC	immunohistochemistry
KRAS	Kirsten rat sarcoma
LD	Linear dichroism
LKB1	Liver kinase B1
lncRNA	Long non coding RNA
MAPK	Mitogen-activated protein kinase
MDA	Minimally differentiated mucinous carcinoma
MDPI	Multidisciplinary Digital Publishing Institute
MEK	MAPK/ERK kinase
MMR	Mismatch repair
mTOR	Mammalian target of rapamycin
MUC6	MUCIN 6
NEF	Neighboring Enhancer of FOXA2 (lncRNA NEF)
NHPVA	Non-HPV-Associated
NRAS	Neuroblastoma RAS viral oncogene homolog
NTRK	Neurotrophic receptor tyrosine kinase
NTRK3	Neurotrophic receptor tyrosine kinase 3
P16	P16INKA4a
Pap test	Papanicolau test
PAX8	Paired box gene 8
PD-L1	Programmed death ligand 1
PI3K	Phosphatidylinositol-3-kinase
PIK3CA	Phosphatidylinositol-4,5-bisphosphate 3-kinase catalytic subunit alpha
POLE	DNA Polymerase epsilon
PR	Progesterone receptor
pRb	Retinoblastoma protein
PTEN	Phosphatase and tensin homolog
RAF	Rapidly accelerated fibrosarcoma kinase
RAS	Rat sarcoma
RB	Retinoblastoma
SMARCA4	SWI/SNF-related, matrix-associated, actin-dependent regulator of chromatin, subfamily A, member 4
STK11	Serine / threonine kinase 11
SWI/SNF	Switch / sucrose non-fermenting
TGF-β	Transforming growth factor beta
TP53	Tumor protein p53
TRK inhibitors	Tropomyosin receptor kinase inhibitors
WHO	World Health Organization
WNT	Wingless-related integration site
